# Building an integrated serosurveillance platform to inform public health interventions: Insights from an experts’ meeting on serum biomarkers

**DOI:** 10.1371/journal.pntd.0010657

**Published:** 2022-10-06

**Authors:** Kirsten E. Wiens, Barbara Jauregui, Benjamin F. Arnold, Kathryn Banke, Djibril Wade, Kyla Hayford, Adriana Costero-Saint Denis, Robert H. Hall, Henrik Salje, Isabel Rodriguez-Barraquer, Andrew S. Azman, Guy Vernet, Daniel T. Leung, on behalf of the Collaboration on Integrated Biomarkers Surveillance

**Affiliations:** 1 Department of Epidemiology, Johns Hopkins Bloomberg School of Public Health, Baltimore, Maryland, United States of America; 2 Mérieux Foundation USA, Washington, District of Columbia, United States of America; 3 Francis I. Proctor Foundation, University of California, San Francisco, California, United States of America; 4 Department of Ophthalmology, University of California, San Francisco, California, United States of America; 5 Bill & Melinda Gates Foundation, Seattle, Washington, United States of America; 6 Institut de Recherche en Santé, de Surveillance Epidémiologique et de Formation (IRESSEF), Dakar, Senegal; 7 International vaccine access center (IVAC), Department of International Health, Johns Hopkins Bloomberg School of Public Health, Baltimore, Maryland, United States of America; 8 Division of Microbiology and Infectious Diseases, National Institute of Allergy and Infectious Diseases (NIAID), Bethesda, Maryland, United States of America; 9 Department of Genetics, University of Cambridge, Cambridge, United Kingdom; 10 Division of HIV, Infectious Diseases, and Global Medicine, University of California, San Francisco, California, United States of America; 11 Division of Experimental Medicine, University of California, San Francisco, California, United States of America; 12 Médecins Sans Frontières, Geneva, Switzerland; 13 Institute of Global Health, Faculty of Medicine, University of Geneva, Geneva, Switzerland; 14 Institut Pasteur de Bangui, Bangui, Central African Republic; 15 Division of Infectious Diseases, Department of Internal Medicine, University of Utah, Salt Lake City, Utah, United States of America; 16 Division of Microbiology and Immunology, Department of Pathology, University of Utah, Salt Lake City, Utah, United States of America; Minia University, EGYPT

## Abstract

The use of biomarkers to measure immune responses in serum is crucial for understanding population-level exposure and susceptibility to human pathogens. Advances in sample collection, multiplex testing, and computational modeling are transforming serosurveillance into a powerful tool for public health program design and response to infectious threats. In July 2018, 70 scientists from 16 countries met to perform a landscape analysis of approaches that support an integrated serosurveillance platform, including the consideration of issues for successful implementation. Here, we summarize the group’s insights and proposed roadmap for implementation, including objectives, technical requirements, ethical issues, logistical considerations, and monitoring and evaluation.

## Introduction

Infectious diseases remain a major cause of morbidity and mortality worldwide. In 2019, 3.68 million deaths were attributable to tuberculosis and other respiratory infections, 1.75 million to enteric diseases, and 747,000 to malaria and neglected tropical diseases (NTDs) [1]. The majority of this burden falls on low- and middle-income countries (LMICs) [1]. The global spread of Severe Acute Respiratory Syndrome Coronavirus 2 (SARS-CoV-2) has further shown how all countries are deeply vulnerable to emerging and reemerging infectious threats. Routine surveillance is a critical component of mitigating spread of these pathogens and depends largely on clinical and microbiological confirmation of infected individuals that seek testing or care. While these tools are valuable for identifying symptomatic cases, they say little about asymptomatic or nonmedically attended infections or the population-level immune landscape. Serological surveys using biomarkers that measure immune responses in serum (i.e., serosurveillance), combined with advances in computational modelling, provide an opportunity to bridge this gap [2,3].

The detection of immune responses in serum has been used for many years, but technological advances are transforming serosurveillance into a powerful tool for epidemiology, mathematical modeling, and public health program design. Sero-epidemiology has guided vaccination strategies for measles and rubella [4], informed vector-control strategies to reduce transmission of malaria [5], and guided tetanus elimination programs [6]. Immunological biomarkers have been used to quantify community exposure to a broad range of pathogens, from arboviruses such as dengue and chikungunya [7,8] to diarrheal diseases such as cholera [9]. Antibodies against vector salivary proteins may also be useful for estimating human exposure to vector bites [10,11] and comparing the efficacy of different vector control strategies [12–14].

Despite the utility of serosurveillance, the costs and logistical challenges involved are prohibitive for comprehensive implementation, particularly in low-resource settings. Advances in multiplex technology for measuring the seroprevalence of multiple pathogens simultaneously could help overcome these barriers [15] as the cost of adding antigens to a multiplex assay is small compared to the cost of collecting specimen [15]. While multiplex assays have been developed and used in serosurveillance for a broad range of pathogens (Table 1), these technologies are still relatively new compared to ELISA and functional antibody assays. Reference sera for a limited number of pathogens are available through the National Institute for Biological Standards and Control (https://www.nibsc.org/), but there is additional need for standardized panels of multiplex beads for different pathogens, populations, and use-cases.

**Table 1:** Published studies for different use-cases of sero-epidemiology by pathogen and source of infection. Pathogens that could be considered for an integrated platform are listed and grouped by primary source of infections. Ways in which sero-epidemiology has previously been used in surveillance of each pathogen are indicated and accompanied by published examples, including both reviews and primary research articles. The numbers in the table indicate references and the gaps illustrate research or surveillance use-cases where serology has not been applied.

Primary source of infection	Pathogen for consideration in an integrated platform	Incidence rate estimates from cross-sectional data	Cumulative infection rate estimates (lasting/saturating Abs)	Vaccine vs. natural infection potentially discernible	Cross-sectional correlates of protection	Used for confirming elimination	Multi-pathogen surveillance via multiplex bead assays
Blood and/or other bodily fluids	*Chlamydia trachomatis*		[23]			[24]	[15,17,25,26]
	Ebola virus						[15]
	Hepatitis B virus			[2]	[27]		
	Hepatitis C virus						
	HIV	[28–32]					[15]
	*Neisseria meningitidis*						
Food, water, and/or soil	*Campylobacter jejuni*	[33,34]	[35,36]				[35]
	*Clostridium tetani*		[37]	[2]	[38]	[19]	[6,15,17]
	*Cryptosporidium parvum*		[35]				[15,17,35]
	Enterotoxigenic *Escherichia coli*		[35]				[35]
	*Giardia intestinalis*		[35]				[15,17,35]
	Hepatitis A virus		[39]		[27]		
	Hepatitis E virus	[40]					[41]
	Lassa virus						[15]
	Norovirus		[35]				[35]
	Poliovirus				[42]		
	*Salmonella enterica* serotype enteriditis	[43,44]	[35]				[15,35]
	*Salmonella enterica* serotype typhimurium		[45]				[15]
	*Schistosoma haematobium*						[46]
	*Schistosoma mansoni*		[47]			[48]	[15,20,46]
	*Shigella*						
	*Strongyloides stercoralis*					[49]	[15,17,20]
	*Taenia solium*						[15,17]
	*Toxoplasma gondii*	[50]	[51]				[17]
	*Vibrio cholerae*	[9,52]			[53,54]		[35]
Respiratory droplets and/or aerosols	*Bordetella pertussis*	[55]			[27,56]		[15]
	*Corynebacterium diphtheriae*		[57]		[57]		[15,17]
	*Haemophilus influenzae* B				[27,38]		
	Measles		[2]		[2]		[15,17]
	Mumps		[58]		[58]		[15,17]
	Respiratory syncytial virus						
	Rhinoviruses						
	Rubella		[2]		[2]		[15,17]
	SARS-CoV-2			[59]			[60,61]
Arthropod vectors	Chikungunya virus	[62]	[7,8]		[63]		[15,64]
	Crimean-Congo hemorrhagic fever virus						[15]
	Dengue virus	[65,66]	[67]				[15]
	Mayaro virus	[68]	[67]				[68]
	*Onchocerca volvulus*					[48]	[15]
	*Plasmodium falciparum* and *Plasmodium vivax*	[5,69,70]					[15,17,20]
	Vector saliva antigens						
	*Wuchereria bancrofti* and *Brugia malayi*					[48]	[15,17,20]
	Yellow fever virus						
	Zika virus		[64,67]				

To harness the full potential of multiplex technologies and create opportunities to shift from vertical programs to program delivery that is coordinated across health risks [16], serological sample collection and analysis must be integrated into surveillance systems as an additional routinely collected source of data used to inform public health decision making. To help overcome the many challenges inherent in establishing new public health systems, a “platform” or global network of public health scientists and practitioners could be created to support the development of and knowledge sharing between locally led integrated serosurveillance programs.

Given the momentum built by these technological advances, a group of approximately 70 scientists gathered in Annecy, France in July 2018 for an Expert Meeting on sero-epidemiology organized by the Mérieux Foundation USA. This group, the Collaboration on Integrated Biomarkers Surveillance (CIBS), included scientists from Australia, Belgium, Cameroon, China, Ethiopia, France, Mozambique, the Netherlands, Norway, Senegal, Spain, Sweden, Switzerland, United Kingdom, United States, and Zambia (see S1 Acknowledgments). CIBS conducted a landscape analysis of existing technologies and approaches that support developing an integrated serosurveillance platform. Based on the results, CIBS established the objectives, technical requirements, ethical issues, logistical considerations, and funding that would be needed for such a platform. Here, we summarize CIBS’s insights and their proposed roadmap for implementation. Many of the areas for development overlap with recommendations released in 2020 by the Pan American Health Organization (PAHO) for integrative serological surveillance in the Americas based on case studies in Mexico and Paraguay [17], as well as a 2021 review on elimination surveillance for NTDs [18].

### Objectives of an integrated serosurveillance platform

The objectives of an integrated serosurveillance platform are 2-fold (Box 1). First, to identify use-cases for serosurveillance (e.g., identifying recent exposure versus immunity; see Box 2 for examples) and support the validation of serological biomarkers markers for each. Second, to support the development of integrated serosurveillance systems, using these biomarkers, to provide actionable health outcome measures for interventions.

**Box 1. Objectives of an integrated platform**

**Specific objectives of an integrated platform**

**1) Define use-cases and identify serological biomarkers**
  - Define use-cases for different pathogens and objectives
  - Identify potential biomarkers for each use-case, including published and experimental biomarkers
  - Define minimum characteristics of appropriate biomarker validation studies
  - Maintain biorepositories of and/or access to international standards (reference reagents) that can be used during biomarker development
**2) Develop serosurveillance systems**
  - Provide support to countries in identifying funding sources and supplies
  - Provide guidance for setting up immunological assays
  - Provide feedback and protocols for designing a sampling frame, conducting community advocacy, and demographic and clinical data collection
  - Provide feedback on organizing logistics and transportation
  - Support development of analytical frameworks for integrating serological and epidemiologic data to translate test results into actionable information

**Box 2. Examples of use-cases for integrated serosurveillance**

**Examples of use-cases for integrated serosurveillance**

**1) Detect recent infection**
  - Example: We want to understand how many children in our district have been exposed to a panel of enteric infections in the past 12 months, and what the infection detection and fatality ratios are based on reported cases and deaths.
  - Approach: If we have validated serum biomarkers that are predictive of recent exposure to the pathogens of interest, we can estimate these metrics using a cross-sectional serosurvey of the known population-at-risk.
**2) Assess cumulative exposure**
  - Example: We want to estimate transmissibility of a panel of pathogens, which are associated with lasting or saturating antibody responses, in a population where vaccines for these pathogens have not been used.
  - Approach: If we have validated serum biomarkers, we can use age-stratified cross-sectional data to estimate the average age of infection, force of infection, R_0_, and/or how much of the population is infected by a given age.
**3) Estimate population susceptibility**
  - Example: We want to know whether a particular segment of the population is protected from infection with a panel of common viral infections due to maternal antibodies, natural infection, and/or vaccination.
  - Approach: If we have known serum biomarkers that are predictive of protection from future infections, we can estimate the fraction susceptible using a cross-sectional survey of the known population-at-risk.

To effectively meet these objectives, the platform should include national, regional, and international components. Country ownership based on locally accepted practices and public health priorities should be the foundation of the design and implementation of the serosurveys, while biomarkers for specific pathogens and use-cases would need to be validated at the regional or international levels. Based on experiences from countries in implementing the initial integrated serosurveillance systems (for example, pilot projects in Cambodia [19], Kenya [20], Nigeria [21], and Vietnam [22]), generic models could be created that countries could adapt to their national priorities and needs. This could include standard guidelines, operating procedures, training modules, as well as a network of technicians in LMICs that could fix and calibrate multiplex machines when needed, and would create a global avenue for interplatform collaboration and exchange of experiences and practices (Box 1).

Ultimately, the platform would serve as a public health resource for sero-epidemiology that informs vaccine campaigns, prophylactic treatments, and other infection control strategies focused on improving the health of the most vulnerable populations. By providing information on a regular basis, it could also enable monitoring the impact of these programs.

### Pathogens

Depending on use-cases, the platform could test biomarkers that measure seroprevalence or recent exposure for a broad range of blood-borne, enteric, respiratory, and vector-borne infections (Table 1). For some pathogens, serological biomarkers may additionally be useful for estimating incidence rates, cumulative infection rates, and correlates of protection, among other applications (Table 1 and Box 2). The performance characteristics (sensitivity and specificity) of relatively few serological markers for serosurveillance have been established to date, as illustrated by the gaps in Table 1. Therefore, initial versions of the platform would include validated and experimental markers. Priority pathogens and use-cases would be determined by implementing countries, which may be influenced by a variety of factors specific to the local context, such as the estimated burden based on hospital case counts, prevalence of key risk factors, and/or whether there are interventions (vaccines, treatments, etc.) that could feasibly be implemented if burden is found to be high in particular populations through serosurveillance. Multiplex bead assays would provide the flexibility to support this, as mixing and matching pathogen-specific beads may be appropriate in many settings. However, having some validated multipathogen panels of multiplex bead assays will also be important here, especially when there are potentially homologous or cross-reactive antigens in the set of pathogens of interest.

### Study population

The study population will also depend on specific pathogens and use-cases (e.g., estimating force of infection, seroprevalence, or population susceptibility; see Box 2 and Table 1 for examples). This is an area where an integrated platform would be instrumental for providing guidance and sharing expertise. For example, to estimate incidence rates for endemic pathogens that infect individuals from a young age, such as many NTDs and enteric pathogens, measuring serological responses in children may be important to capture differences age-specific seroprevalence that might plateau in older age-groups [15,35]. In contrast, teens and adults are more relevant for serosurveillance of pathogens such as HIV, with efforts to sample high-risk groups that may be less likely to be sampled in traditional study designs [15]. For integrated surveillance of pathogens that require measurements in different age groups, initial population-based surveys could be conducted across a wide age range, followed by more targeted, adaptive surveys that focus on disease- or program-specific use-cases.

Timing of surveys would also depend on the biomarkers included and specific use-cases. An annual survey would be sufficient for studying long-lasting antibody responses to pathogens such as measles or rubella, while biannual surveys would be required for antibodies with shorter life such as *Vibrio cholerae* and other enteric pathogens. For vaccine-preventable diseases, immunization schedules or campaigns would also need to be taken into consideration.

### Community and stakeholder engagement

Identifying all relevant community stakeholders and engaging with them is critical for the successful implementation of any new program, which have been illustrated through examples from the Ebola response in West Africa [71]. Effective community and stakeholder engagement (CSE) requires dedicated funding, sponsor commitments, and moral support. It requires engaging a broad range of individual stakeholders, including women, frontline healthcare workers, and teachers, among many others, who may not be all represented on, for example, community advisory boards. Engagement needs to emphasize listening to stakeholders, identifying opportunities for deliberation, and developing relationships and conditions needed to integrate the program into existing health infrastructure, where results warrant. Critically, it requires making sure that the research has a direct and timely impact on public health programs in the surveyed community. Finally, any CSE strategy needs to be implemented in a way that allows for meaningful evaluation of its effectiveness.

### Ethical considerations

Collecting biological specimen and sociodemographic data for research purposes requires careful ethical review and clearance through institutional review boards. Therefore, these guidelines are already in place in existing survey systems (e.g., Demographic and Health Survey, Malaria and AIDS Indicator Surveys). Rules for ownership of data and specimen should also be established, with efforts made to process specimen locally within the implementing country when possible and to build this capacity when it is not. Intellectual property on research conducted as part of the program must consider rights of the countries from which the specimens originate and provide guarantees that any analytical and/or laboratory surveillance tools derived from the research will be made available in the country.

### Specimen collection and testing

The most appropriate specimen will depend on the scale of the survey and resources available. While saliva is the most practical specimen for large epidemiological surveys, oral fluid assays have historically had lower sensitivity than comparable blood-based assays. Although, a recent study showed that saliva-based tests have similar performance to plasma-based tests for SARS-CoV-2 [60,72], suggesting that utility of saliva-based tests may be pathogen-specific. Overall, blood remains the most reliable specimen for biomarker detection. However, venipuncture is an invasive procedure that requires specific training, generates substantial biohazardous waste, and requires transporting blood tubes safely in below zero conditions. Collecting capillary blood through dried blood spots (DBS) provide a more scalable alternative. DBS show comparable antibody measurements to serum samples for falciparum malaria, some bacterial and protozoal pathogens, and numerous viral pathogens, including vaccine-preventable diseases [73].

DBS also provide flexibility in testing locations. DBS may be used on site in rapid lateral-flow assays. DBS can be transported to remote sites for testing with more resource-intensive methods such as multiplex immunoassays. They can be kept at cool or ambient temperatures for several weeks before they are frozen for long-term storage [73], as long as high temperatures are avoided. Serum Separator cards can be used to automatically separate serum from DBS, further reducing the effort required to process these samples [74].

While DBS allow several markers to be tested from a low volume of blood within a single sample, there is no standard platform or procedure for running and vetting results from multiplex antibody assays using DBS. In addition, platforms such as Luminex that are used for running multiplex assays are often only available in national or regional labs and require regular calibration and use of positive controls for consistency. Both individual and multiplex assays will need to be compared and validated before use in an integrated platform, including comparisons between DBS and venous blood samples. The choice of the platform itself will depend on the type of support available in-country. For example, Luminex’s MAGPIX system is a robust system that can often be repaired without the need for trained technicians, who are often not available in LMICs. Importantly, these protocols and methods should be shared through the platform’s network, especially as new technologies (e.g., rapid or point-of-care tests, microarrays, fieldable instruments, phage-display approaches) become available.

### Logistics and resources

As described above, there will be various logistical challenges in implementing new serological surveys. Central reference labs could help with adoption, dissemination, and local capacity building. Public–private initiatives could also be leveraged. Within Africa, engaging the Africa CDC and WHO-AFRO will be important to ensure shared vision across the continent. Importantly, care should be taken that these efforts do not divert budgets and skilled technicians from the healthcare system.

One way to address logistical challenges is to integrate the platform within existing active and passive surveillance systems [17,18]. Existing surveys that could be leveraged to accommodate multiplex testing include the Demographic and Health Survey, Malaria and AIDS Indicator Surveys, and NTD transmission assessment surveys [15], though the latter are often targeted to narrow geographic units and ages. Another potential source is remnants of samples from routine blood draws, which enabled rapid estimating of SARS-CoV-2 seroprevalence in areas where these were available [75], further highlighting the utility of an integrated serosurveillance system. The most appropriate survey or surveillance tool will depend on timing of the surveys within each country and, importantly, on continued sources of funding.

The CIBS and an International Coordinating Committee (ICC) could also provide guidance and oversight through coordination with a National Survey Program (NSP) (Box 3). In this framework, the NSP could be part of the Ministry of Health and intersect with national statistical agencies, or representatives from them, where this support is needed. It would coordinate all activities in country and liaise with survey staff and researchers. Survey staff would include community relays or public health workers (participant recruitment, demographic questionnaires, GPS, incentives distribution, logistics, and feedback), community health workers (specimen collection, participants information, and feedback), and regional and central laboratory personnel. Support from local health authorities would be critical for this type of program, and funding would need to be secured at international and national levels [16], with comprehensive roadmaps developed, including paths to sustainability.

**Box 3. Functions of an international coordinating committee**

**Functions of the International Coordinating Committee (ICC)**

✓ Oversight of program;
✓ Mobilize scientific expertise;
✓ Prepare program documentation;
✓ Secure funding;
✓ Provide guidance for assay validation;
✓ Oversee quality control activities;
✓ Centralize and share data;
✓ Centralize and dispatch specimens to specialized and research labs;
✓ Coordinate data analysis;
✓ Establish general and country/community-specific guidelines;
✓ Provide feedback to national disease programs;
✓ Organize international communication and dissemination of results.

In addition to logistical challenges described above, appropriate supply chains need to be developed to ensure availability of data collection and transport devices, including their transport into communities. National public health labs could take the lead in these efforts. Since purchasing these tools may be difficult in many settings, existing resources should be evaluated, strengthened, and used, where possible. When these tools are not available, alternative solutions/organizations could be identified through the NSP. This is another area where an integrated platform could provide critical support by creating opportunities for resource sharing between existing programs.

### Monitoring and evaluation

Monitoring and evaluation are critical components of any new surveillance program to ensure effective use of resources. We propose 2 key areas for evaluation. First, pilot studies to assess the feasibility of a new platform, including proficiency testing with blinded test samples. Among criteria considered should also be the degree of community knowledge regarding disease prevention and intervention. Second, analyses of whether results from integrated surveillance studies led to a change in policy (e.g., whether a new program was started or changed, whether it triggered an intervention, or changed a clinical diagnosis) and whether it helped improve understanding of disease patterns. Long term, changes in disease patterns and reductions in disease burden should be evaluated (for example, as was done in Zambia to evaluate the effect of targeted indoor residual spraying on malaria incidence [76]).

As sero-epidemiology is resource-intensive, an additional area for evaluation is cost-effectiveness of preventing outbreaks. The potential savings of identifying immunity gaps and tailoring interventions should be modelled to evaluate whether investment in, for example, additional vaccination, would be a better use of funds.

### Advocacy

Local advocacy will be essential, with efforts made to effectively make use of advocacy resources that already exist. Guidelines regarding communicating data that are considered sensitive by local and national authorities need to be established. Potential stigmatization of communities that fail to efficiently implement interventions should be considered. For advocacy efforts to be successful, they must actively engage Ministries of Health, national disease programs, political authorities, community leaders, civil society, and religious authorities.

When disease burden reduction has been achieved, it may be difficult to justify asking health authorities and communities to give blood or to spend additional limited resources. Thus, the monitoring and evaluation approaches described above will be critical to evaluate the continued utility of an integrated platform and advocate for funding as needed.

Evidence-based arguments supporting the efficiency and cost-effectiveness of integrated surveys will also support advocacy internationally. Given the global interrelatedness of old and new emerging infectious diseases, there is a critical need to have well-coordinated responses [77]. In this capacity, WHO plays an essential role supporting national public health programs. Partners such as the Mérieux Foundation, the Global Fund, Gavi, the Vaccine Alliance, and the Bill and Melinda Gates Foundation should work with WHO with the primary aim to leverage and strengthen local and regional networks and partners to further strengthen public health laboratory performance. Importantly, any support for these programs and networks must prioritize knowledge production and use within the implementing countries [78].

### Areas for innovation

Innovation is needed at several levels to successfully implement an integrated platform, as is research funding to support these efforts. Technologically, new biomarkers, existing biomarkers (e.g., Table 1), and combinations of biomarkers need to be identified and validated. It will also be important to evaluate the best specimen for broad application (e.g., DBS or saliva), including new devices that reduce pain, increase acceptance by participants, and improve ease of storage and transportation. These devices must address multiparameter testing from a single specimen, safe storage in degraded conditions (temperature, dust, moisture), space in transport packages, and cost.

Innovation is also needed to address logistical and resources issues. New technologies such as drones may be useful to transport specimen and supplies to and from remote locations [79]. Existing transportation capacities such as commercial companies involved in persons or goods transportation, pharmaceutical distribution, or other surveillance schemes should be evaluated.

Innovation in study design and analysis is also critical for defining pathogen priorities and sampling frames, as well as providing clear results and recommendations to disease programs. As in any survey, it will be important to carefully consider epidemiological components (e.g., age, sex, family environment, geographical environment, sample size, and GPS). For an integrated platform, study designs must also be harmonized and optimized across diverse disease and surveillance priorities. Analysis pipelines that create informative results from a single assay for various diseases need to be developed, such as disease burden maps that overlay high-burden populations for multiple pathogens simultaneously. Digital health solutions could help overcome some of these challenges. For example, tools like rapid diagnostic tests linked to cloud servers [80] could be developed for sending test results to a national dashboard, providing real-time disease maps and trends to health authorities.

## Conclusions

Serosurveillance systems that monitor many pathogens simultaneously and that are integrated with established mechanisms of data collection and analysis—with support from a platform of local, regional, and global collaborators—have the potential to dramatically improve our understanding of disease burden and support more effective public health decision-making. In this paper, we have described gaps that must be filled for such a platform to be successful, from rigorous validation of serological assays to community partnerships and development of novel analytical frameworks. The current COVID-19 pandemic has enabled advancement in many of these areas [81]. It has also highlighted the importance of integrating serological with traditional surveillance data across human and animal health programs for preventing and controlling disease emergence. Given this momentum and the importance of integrated surveillance systems for responding to future infectious threats, the time is now to move forward with filling in the remaining gaps. Ultimately, this will enable better, more comprehensive data that can be used for designing interventions to reduce the burden of endemic and emerging diseases.

## Supporting information

S1 Acknowledgments Membership of the Collaboration on Integrated Biomarkers Surveillance. (DOCX)
