# Supplementary material for: Building an integrated serosurveillance platform to inform public health interventions: Insights from an experts’ meeting on serum biomarkers
Source: PLoS Negl Trop Dis. 2022 Oct 6;16(10):e0010657. doi: 10.1371/journal.pntd.0010657 (PMC9536637; doi:10.1371/journal.pntd.0010657)
Supplement: S1 Acknowledgments — (DOCX) [file pntd.0010657.s001.docx]

# **Building an integrated serosurveillance platform to inform public health interventions: Insights from an experts’ meeting on serum biomarkers**

## Acknowledgments

The following are members of the Collaboration on Integrated Biomarkers Surveillance, who attended the meeting titled, “Expert Meeting on a platform for biomarker-based surveillance in communities to guide disease control interventions,” on July 25 to 27, 2018, at Les Pensieres Center for Global Health, Veyrier-du-Lac, France: Jon Andrus (George Washington University, Washington, DC, USA), Ben Arnold (UC Berkeley, Berkeley, CA, USA), Lawrence Ayong (Centre Pasteur du Cameroun, Yaoundé, Cameroon), Andrew Azman (Johns Hopkins University, Baltimore, MD, USA), Christine Bain (Advanced Biosciences Laboratory, Lyon, France), Kathryn Banke (Bill and Melinda Gates Foundation, Seattle, WA, USA), Bob Black (Johns Hopkins University, Baltimore, MD, USA), Sarah Browne (FDA, Silver Spring, MD, USA), Juliet Bryant (Fondation Mérieux, Lyon, France), AC Camacho (DTRA, Washington, DC, USA), Jean-Sebastien Casalengo (Université Claude Bernard, Lyon, France), Adriana Costero Saint Denis (NIH, Bethesda, MD, USA), Jane Cunningham (WHO, Geneva, Switzerland), Sabine Dittrich (FIND, Geneva, Switzerand), Marc Essodaigui (Advanced Biosciences Laboratory, Lyon, France), Alison Evarts (Merieux Foundation, Washington, DC, USA), Matthew Ferrari (Penn State University, University Park, PA, USA), Lia Florey (USAID, Washington, DC, USA), Dean Garrett (ICF, Silver Spring, MD, USA), Françoise Gay-Andrieu (bioMérieux, Marcy-l'Etoile, France), Bryan Greenhouse (UCSF, San Fransisco, CA, USA), Robert Hall (NIH, Bethesda, MD, USA), Kyla Hayford (Johns Hopkins University, Baltimore, MD, USA), Shelley Hossenlopp (Spot-On-Sciences, Austin, TX, USA), Rolf Kramer (ECDPC, Stockholm, Sweden), Daniel Leung (University of Utah, Salt Lake City, UT, USA), Nafissatou Leye (IRESSEF, Dakar, Senegal), Francisco Luquero (Epicentre, Geneva, Switzerland), Ivalda Macicame (NIH, Maputo, Mozambique), Yuka Manabe (Johns Hopkins, Baltimore, MD, USA), Henshaw Mandi (CEPI, Oslo, Norway), Christine Markwalter (Duke University, Durham, NC, USA), Anne Martin (Akros, Lusaka, Zambia), Elhadji Mbaye (IRESSEF, Dakar, Senegal), Souleymane Mboup (IRESSEF, Dakar, Senegal), David McGregor (LSHTM, London, UK), Martin Mengel (GMX, Valencia, Spain), Mark Miller (NIH, Bethesda, MD, USA), Michael Mina (Harvard School of Public Health, Cambridge, MA, USA), Marie Moroso (Fondation Mérieux, Lyon, France), Ivo Mueller (The Walter and Eliza Hall Institute of Medical Research, Parkville, Australia), Abdoulaye Nikiema (ASLM, Addis-Abeba, Ethiopia), Berthe Marie Njanpop (Paris, France), Tom Nutman (NIH, Rockville, MD, USA), David Olson (WHO, Geneva, Switzerland), Emily Penrose (Merieux Foundation USA, Washington, DC, USA), Jessica Radzio-Basu (Penn State University, University Park, PA, USA), Olivier Raynaud (Bill and Melinda Gates Foundation, Seattle, WA, USA), Franck Remoue (IRD, Montpellier, France), Isabel Rodriguez-Barraquer (UCSF, San Fransisco, CA, USA), Kolawole Salami (CEPI, Oslo, Norway), Amadou Sall (Institut Pasteur, Dakar, Senegal), Richard Schoske (DTRA, Washington, DC, USA), William Evan Secor (CDC, Atlanta, GA, USA), Yvan Sergeant (Quanterix, Belgium), Shash Shashidhar (Penn State University, University Park, PA, USA), Daniel Sikkema (Quanterix, USA, Lexington, MA, USA), Benjamin Svarczkopf (Luminex, Austin, TX, USA), Kay van der Horst (Penn State University, University Park, PA, USA), Fiona van der Klis (RIVM, Utrecht, the Netherlands), Jessica Vanhomwegen (Institut Pasteur, Paris, France), Guy Vernet (Merieux Foundation USA, Washington, DC, USA), Djibril Wade (IRESSEF, Dakar, Senegal), Joseph Wu (School of Public Health, The University of Hong Kong, Hong Kong SAR, China), Lindsey Wu (LSHTM, London, UK)
